# Non-contrast quantitative T1-mapping indicates that salvaged myocardium develops edema during coronary occlusion, whereas infarction exhibits evidence of additional reperfusion injury

**DOI:** 10.1186/1532-429X-13-S1-O63

**Published:** 2011-02-02

**Authors:** Martin Ugander, Micheas Zemedkun, Li-Yueh Hsu, Abiola J Oki, O Julian Booker, Peter Kellman, Andreas Greiser, Anthony H Aletras, Andrew E Arai

**Affiliations:** 1 National Institutes of Health, Bethesda, MD, USA; 2 Siemens AG Healthcare Sector, Erlangen, Germany

## Introduction

T2-weighted cardiovascular magnetic resonance (CMR) can characterize myocardial edema in acutely infarcted myocardium and in recently ischemic but salvaged myocardium in ways that are distinct and complementary to late gadolinium enhanced (LGE) images. If T2 can be used to detect myocardial edema, then non-contrast T1 quantification may also be abnormal in a similar fashion.

## Purpose

To quantitatively assess if salvaged myocardium develops edematous changes during coronary occlusion, but not reperfusion injury as can be seen in infarction.

## Methods

Dogs (n=9) underwent coronary occlusion and reperfusion to delineate the time course of development of changes in non-contrast T1 abnormalities. T1 was quantified by 1.5T CMR (Siemens) using a Modified Look-Locker Inversion Recovery (MOLLI) sequence. T2-prepared SSFP (T2p) and LGE images were acquired at the end of the experiment to define 1) Infarction [LGE positive], 2) Salvaged myocardium [T2p positive and LGE negative] and 3) Remote [T2p and LGE negative]. Myocardium which ultimately ended being classified into these three tissue types was identified, and T1 was quantified over time starting from baseline prior to occlusion.

## Results

**Figure 1:**
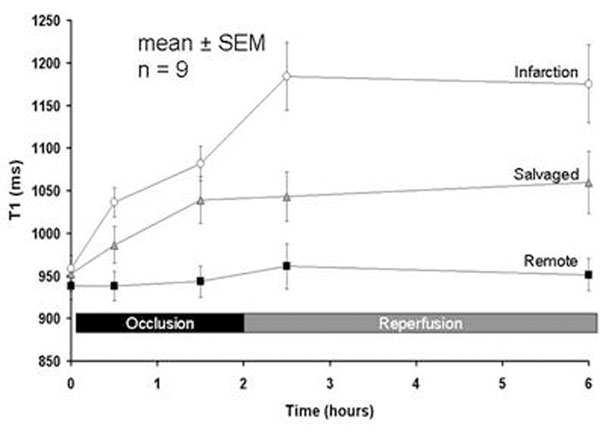
Time course of non-contrast T1 values during occlusion and reperfusion for myocardium which ultimately will become infarcted, salvaged and remote.

See Figure 1. During occlusion, T1 increased in tissue which ultimately became Infarction or Salvaged (p<0.01 for both), and to a greater extent in tissue which ultimately became Infarction compared to Salvaged (p=0.03). Within 30 minutes of reperfusion, T1 of Infarction increased further (p=0.001), whereas T1 of Salvaged myocardium remained unchanged after reperfusion (p=0.73).

## Conclusions

Coronary occlusion leads to an increase in non-contrast T1, consistent with edema, both in tissue which ultimately will become infarcted, and in tissue which ultimately will be salvaged. The increase in T1 during occlusion is more pronounced, consistent with more severe ischemia, in tissue which ultimately will become infarcted compared to that which becomes salvaged. Following reperfusion, only the tissue which becomes infarcted, but not that which becomes salvaged, increases additionally in T1, and this is consistent with reperfusion injury in infarcted myocardium.

